# Effect of Type 2 Diabetes Mellitus on the Hypoxia-Inducible Factor 1-Alpha Expression. Is There a Relationship with the Clock Genes?

**DOI:** 10.3390/jcm9082632

**Published:** 2020-08-13

**Authors:** Carolina López-Cano, Liliana Gutiérrez-Carrasquilla, Ferran Barbé, Enric Sánchez, Marta Hernández, Raquel Martí, Vicky Ceperuelo-Mallafre, Mireia Dalmases, Sonia Fernández-Veledo, Joan Vendrell, Cristina Hernández, Rafael Simó, Albert Lecube

**Affiliations:** 1 Endocrinology and Nutrition Department, University Hospital Arnau de Vilanova, Obesity, Diabetes and Metabolism (ODIM) Research Group, IRBLleida, University of Lleida, 25198 Lleida, Spain; karolopezc@gmail.com (C.L.-C.); liligutierrezc@gmail.com (L.G.-C.); esanchez@irblleida.cat (E.S.); martahernandezg@gmail.com (M.H.); rmarti@irblleida.cat (R.M.); 2 Respiratory Department, University Hospital Arnau de Vilanova-Santa María, Translational Research in Respiratory Medicine, IRBLleida, Universityof Lleida, 25198 Lleida, Spain; febarbe.lleida.ics@gencat.cat (F.B.); mdalmases.lleida.ics@gencat.cat (M.D.); 3 Centro de Investigación Biomédica en Red de Enfermedades Respiratorias (CIBERES), Instituto de Salud Carlos III (ISCIII), 28029 Madrid, Spain; 4 Endocrinology and Nutrition Department, University Hospital de Tarragona Joan XXIII, Institut d’Investigació Sanitària Pere Virgili (IISPV), Rovira i Virgili University, 43001 Tarragona, Spain; victoria.ceperuelo@urv.cat (V.C.-M.); sonia.fernandezveledo@gmail.com (S.F.-V.); jvo@comt.es (J.V.); 5 Centro de Investigación Biomédica en Red de Diabetes y Enfermedades Metabólicas Asociadas (CIBERDEM), Instituto de Salud Carlos III (ISCIII), 28029 Madrid, Spain; cristina.hernandez@vhir.org; 6 Endocrinology and Nutrition Department, University Hospital Vall d’Hebron, Diabetes and Metabolism Research Unit, Valld’HebronInstitut de Recerca (VHIR), Autonomous University of Barcelona, 08035 Barcelona, Spain

**Keywords:** type 2 diabetes, hypoxia, metabolic control, chronodisruption, clock genes

## Abstract

Limited reports exist on the relationships between regulation of oxygen homeostasis and circadian clock genes in type 2 diabetes. We examined whether the expression of Hypoxia-Inducible Factor-1α (HIF-1α) and HIF-2α relates to changes in the expression of clock genes (Period homolog proteins (PER)1, PER2, PER3, Retinoid-related orphan receptor alpha (RORA), Aryl hydrocarbon receptor nuclear translocator-like protein 1 (ARNTL), Circadian locomotor output cycles kaput (CLOCK), and Cryptochrome proteins (CRY) 1 and CRY2) in patients with type 2 diabetes. A total of 129 subjects were evaluated in this cross-sectional study (48% with diabetes). The gene expression was measured by polymerase chain reaction. The lactate and pyruvate levels were used as surrogate of the hypoxia induced anaerobic glycolysis activity. Patients with diabetes showed an increased plasma concentration of both lactate (2102.1 ± 688.2 vs. 1730.4 ± 694.4 uM/L, *p* = 0.013) and pyruvate (61.9 ± 25.6 vs. 50.3 ± 23.1 uM/L, *p* = 0.026) in comparison to controls. However, this finding was accompanied by a blunted HIF-1α expression (1.1 (0.2 to 5.0) vs. 1.7 (0.4 to 9.2) arbitrary units (AU), *p* ≤ 0.001). Patients with diabetes also showed a significant reduction of all assessed clock genes’ expression. Univariate analysis showed that HIF-1α and almost all clock genes were significantly and negatively correlated with HbA1c concentration. In addition, positive correlations between HIF-1α and the clock genes were observed. The stepwise multivariate regression analysis showed that HbA1c and clock genes independently predicted the expression of HIF-1α. Type 2 diabetes modifies the expression of HIF-1α and clock genes, which correlates with the degree of metabolic control.

## 1. Introduction

In recent years, increasing evidence supports the contribution of type 2 diabetes to the development of sleep breathing disorders [1,2]. Thus, patients with type 2 diabetes experience higher rates of microarousals and an increased sympathetic nerve activity during sleep in comparison to control subjects, together with sleep quality reduction and excessive daytime sleepiness [3,4]. But more important, type 2 diabetes appears as an independent risk factor for severe nocturnal hypoxia [5]. In healthy subjects, hypoxic conditions trigger Hypoxia-Inducible Factor (HIF)-1 gene activation to stimulate angiogenesis and regulate cells to an anaerobic metabolism [6]. This major regulator of oxygen homeostasis is a heterodimer transcription factor that combines a constitutively expressed β-subunit with an oxygen-regulated α-subunit [7]. There are two closely related nonredundant HIF-α subunits (HIF-1α and HIF-2α, the last also called Endothelial PAS Domain Protein 1 or EPAS1) with capacity to activate hypoxia response elements under hypoxia conditions. However, tissues from humans and animals with type 2 diabetes experience an altered cellular response to hypoxia that reduces the relative expression of HIF-1α, modifies glycolytic flux, and affects insulin secretion [8,9].

Our circadian clock, a transcriptional and translational self-regulating feedback loop, is in the suprachiasmatic nucleus. It works as a pacemaker that receives photic information conferring a 24-h structure on processes at all levels, from behavior to gene expression [10]. In fact, clock genes participate in important cellular processes such as cell proliferation or apoptosis in a wide variety of peripheral tissues such as muscle, adipose tissue, liver, and pancreas [11,12]. Although less well-characterized in humans than in rodents, there is clear evidence that metabolic pathways follow circadian rhythms, including glucose and lipid metabolism, energy expenditure, fasting and appetite [13,14]. In this way, the endocrine pancreas has an intrinsic self-sustained clock that controls not only the secretion and signaling of insulin and glucose uptake but also the proliferation and β cells growth [15]. Therefore, changes in the circadian rhythm derived from our lifestyle, such as nighttime work and meals, have also been associated with an increased incidence of metabolic disorders including insulin resistance, type 2 diabetes and excess body weight [16,17].

Until now, there have been limited reports on the relationships between regulation of oxygen homeostasis and circadian clock genes in type 2 diabetes [18,19]. To further increase our knowledge in this emerging field, we have designed a study to examine whether the expression of HIF-α subunits (HIF-1α and HIF-2α) is connected with changes in the expression of eight core circadian clock genes according to the presence of type 2 diabetes.

## 2. Material and Methods 

### 2.1. Statement on Ethics

The human ethics committee of the Arnau de Vilanova University Hospital approved the study (CEIC-1516, 2 June 2016), which was steered according to the ethical guidelines of the Helsinki Declaration. Informed written consent was obtained from all individual participants included in the study.

### 2.2. Design of the Study and Description of the Study Population

The study examined patients of Caucasian origin from March 2018 to October 2018 attending to the outpatient Endocrinology Clinic. Patients that fulfilled the following inclusion criteria were invited to participate in the study: type 2 diabetes with at least 5 years of known duration, age between 18 and 70 years, no medical history of lung disease and/or heart failure, a body mass index (BMI) of less than 40 kg/m^2^, and a nighttime sleep pattern of at least 6 h. Among the 243 patients who met the inclusion criteria, we excluded 114 for the following reasons: unwillingness to participate in the study (*n* = 47), treatment with continuous positive airway pressure (CPAP, *n* = 22), active malignancy or malignancy diagnosed within the previous five years (*n* = 14), autoimmune diabetes including type 1 diabetes and latent autoimmune diabetes in adult (*n* = 12), treatment with drugs with activity on the central nervous system (e.g., hypnotics, antidepressants, sedatives, psycholeptics, anxiolytics; *n* = 9), history of alcohol or caffeine abuse (*n* = 5), work night shifts (*n* = 4), and neuromuscular diseases (*n* = 1). No pregnant or lactating women were included in the study.

### 2.3. Gene expression by Real-Time Quantitative PCR.

After patients fasted overnight for 12 h (last meal before 21 h), venous blood was collected from the antecubital vein. Samples were separated by centrifugation (2000× *g* at 4 °C for 20 min) and peripheral blood mononuclear cells were stored until analysis. One microgram of ribonucleic acid (RNA) was transcribed to complementary deoxyribonucleic acid (DNA) with random primers using deoxynucleotyde (dNTP) Mix (100 mM), MultiScribe™ Reverse Transcriptase (50 U/μL) (Applied Biosystems, Foster City, CA, USA) and RNase Inhibitor using High-Capacity cDNA Reverse Transcription Kit with RNase Inhibitor. Thermal Cycler Conditions were as follows: 25 °C for 10 min, 37 °C for 120 min, 85 °C for 5 min, and 4 °C ∞ according to the manufacturer’s protocol (Cat.No. 4374967 Applied Biosystems, Foster City, CA, USA). GeneAmp^®^ PCR System 9700 Thermal Cycler by Applied Biosystems was used (Applied Biosystems, Foster City, CA, USA).

Quantitative gene expression for HIF-1α, HIF-2α, Period homolog proteins (PER)1, PER2, PER3, Retinoid-related orphan receptor alpha (RORA), Aryl hydrocarbon receptor nuclear translocator-like protein 1 (ARNTL, also called Brain and muscle ARNT-like protein-1 or BMAL1), Circadian locomotor output cycles kaput (CLOCK), and Cryptochrome proteins (CRY)1 and CRY2 genes were evaluated by qPCR on a 7900HT Fast Real-Time PCR System using the TaqMan Gene Expression Assay (Applied Biosystems) based on TaqMan^®^ Assays QPCR Guarantee Program (Applied Biosystems, Foster City, CA, USA) [20] The next Taqman probes were used: Hs00153153_m1, Hs01026149_m1, Hs00242988_m1, Hs00256143_m1, Hs00213466_m1, Hs00536545_m1, Hs00154147_m1, Hs00231857_m1, Hs00172734_m1 and Hs00323654_m1, respectively. Results were calculated using the comparative Ct method (2^−ΔΔCt^), normalized to the expression of the reference gene 18S (Hs 03928985_g1) and expressed relative to a calibrator (a mix of samples). TaqMan™ Fast Advanced (ref 4444557 Applied Biosystems, Foster City, CA, USA) was used. This master mix employs Applied Biosystems™ AmpliTaq™ Fast DNA Polymerase (Applied Biosystems, Foster City, CA, USA), which has been engineered for enhanced stability. Protocol used in 7900HT Fast Real-Time PCR System was as follows: 50 °C for 2 min, 95 °C for 10 min, and 40 cycles of 95 °C for 15 s and 60 °C for 1 min. Thermo Fisher Scientific supports MIQE guidelines provide all information necessary to ensure MIQE compliance when publishing the results of real-time PCR experiments [21]. All assays are inventoried assays and the assay probe spans an exon junction. For 18S (Hs 03928985_g1) assay design, the assay primers and probes lie within a single exon. This assay will detect gDNA.

### 2.4. Circulating Lactate and Pyruvate Measurement

The activity of anaerobic glycolysis was estimated indirectly through the measurement of circulating plasma levels of lactate and pyruvate. Plasma lactate levels were measured using the EnzyFluoTM L-Lactate Assay kit (BioAssay Systems, Hayward, CA, USA). The assay sensitivity was 1 µmol/L and linearity up to 50 µmol/L. Plasma pyruvate levels were measured using the EnzyChrom TM Pyruvate Assay kit (BioAssay Systems, Hayward, CA, USA). The linear detection range was 0.2 to 50 µmol/L.

### 2.5. Statistical Analysis

A normal distribution of the variables was established using the Kolmogorov-Smirnov test, and data were expressed as median (interquartile range), mean ± standard deviation or percentage. The χ^2^ test was used to compare categorical variables, and Student’s *t*-test and Mann-Whitney test were used to compare non categorical data according to their distribution. The relationship between the variables was examined by Pearson’s and Spearman’s correlation tests. A stepwise multivariate regression analysis was performed to explore the variables independently related to HIF-1α expression. Variables significantly associated with its measurement in the bivariate analysis (i.e., glycated hemoglobin (HbA1c) values), together with clinically relevant variables with a potential impact on sleep breathing (i.e., gender, body mass index (BMI), age) were included in the analysis (model 1). Later, clock genes expression was also included (model 2). In a parallel way, 8 more stepwise multivariate regression analyses were done to investigate the variables independently linked with the different clock genes expression. All p values were based on a two-sided test of statistical significance (two-tailed, 95% confidence interval). Significance was accepted at the level of *p* < 0.05. Statistical analyses were performed using the SPSS statistical package for the Social Sciences software (IBM SPSS, Statistics for Windows, Version 20.0. Armonk, NY, USA).

## 3. Results

The main clinical and anthropometric data of the 129 patients that were finally included in the study are displayed in Table 1. Although both groups displayed a similar age, patients with type 2 diabetes showed a higher prevalence of women, a higher degree of obesity, a more atherogenic lipid profile and a higher prevalence of hypertension than controls.

**Figure 1:**
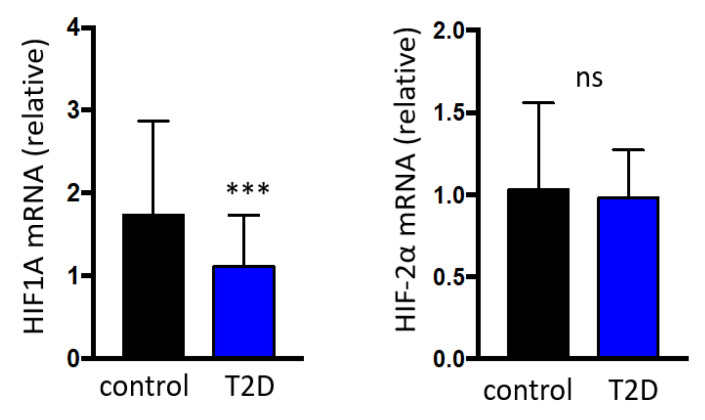
Relative HIF-1α and HIF-2α gene expression based on the presence of type 2 diabetes. Data are presented as median and interquartile. *** *p* < 0.001; ns = non-significant. HIF: Hypoxia-Inducible Factor; T2D: type 2 diabetes; mRNA: messenger ribonucleic acid.

**Figure 2:**
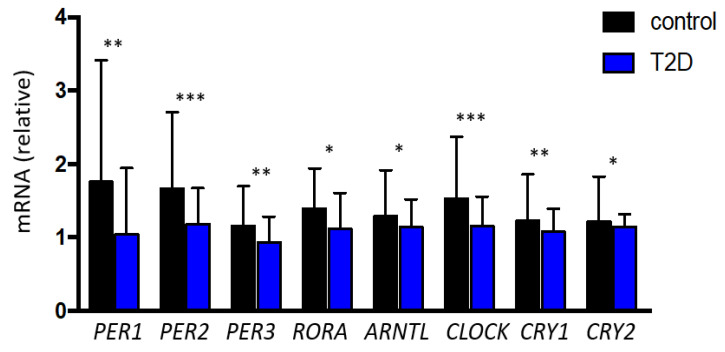
Relative gene expression of the clock genes evaluated in the study according to the presence of type 2 diabetes. Data are presented as median and interquartile. *** *p* < 0.001; ** *p* < 0.01; * *p* < 0.05. PER: Period Homolog Proteins; RORA: Retinoid-related Orphan Receptor Alpha; ARNTL: Aryl hydrocarbon Receptor Nuclear Translocator-Like protein-1; CLOCK: Circadian Locomotor Output Cycles Kaput; CRY: Cryptochrome proteins; T2D: type 2 diabetes.

Patients with type 2 diabetes showed an increased plasma concentration of both lactate (2102.1 ± 688.2 vs. 1730.4 ± 694.4 uM/L, *p* = 0.013) and pyruvate (61.9 ± 25.6 vs. 50.3 ± 23.1 uM/L, *p* = 0.026) in comparison to the control group. However, subjects with type 2 diabetes showed a significantly reduced HIF-1α (1.1 (0.8 to 1.7) vs. 1.7 (1.1 to 2.8) arbitrary units (AU), *p* ≤ 0.001) expression compared to the control group, without differences in HIF-2α expression (0.9 (0.6 to 1.2) vs. 1.0 (0.7 to 1.5) AU, *p* = 0.096) (Figure 1).Patients with type 2 diabetes also exhibited a significant reduction in the expression of all assessed clock genes: PER1 (1.0 (0.7 to 1.9) vs. 1.7 (0.9 to 3.4) AU, *p* = 0.003), PER2 (1.1 (0.7 to 1.6) vs. 1.6 (1.2 to 2.7) AU, *p* < 0.001), PER3 (0.9 (0.6 to 1.2) vs. 1.1 (0.8 to 1.7) AU, *p* = 0.004), RORA (1.1 (0.7 to 1.6) vs. 1.3 (0.9 to 3.0) AU, *p* = 0.032), ARNTL (1.1 (0.7 to 1.5) vs. 1.2 (0.9 to 1.9) AU, *p* = 0.013), CLOCK (1.1 (0.8 to 1.5) vs. 1.5 (1.1 to 2.3) AU, *p* = 0.001), CRY1 (1.0 (0.8 to 1.3) vs. 1.2 (0.9 to 1.8) AU, *p* = 0.003) and CRY2 (1.1 (0.8 to 1.3) vs. 1.2 (0.9 to 1.8) AU, *p* = 0.018) (Figure 2).

In the entire population, univariate analysis showed that expression of HIF-1α but no HIF-2α, as well as clock genes such as PER1, PER2, PER3, RORA, ARNTL, CLOCK, CRY1 and CRY2 were significantly and negatively correlated with HbA1c concentration (Table 2). In addition, positive correlations between HIF-1α gene expression and all the assessed clock genes were also observed (Table 2).

Finally, the stepwise multivariate regression analysis performed in the entire population showed that HbA1c (but not age, gender, or BMI) independently predicted the gene expression of HIF-1α (R^2^ = 0.114) (Table 3). In addition, when clock genes were introduced in the multivariate regression analysis (model 2), PER1, PER2, PER3, CLOCK and CRY2 gene expression (but no age, gender, BMI or HbA1c) independently predicted the expression of HIF-1α (R^2^ = 0.924) (Table 3).

In addition, the stepwise multivariate regression analysis showed that HIF-1α relative expression (but not age, gender, BMI or Hba1c) independently predicted the expression of PER1 (R^2^ = 0.784; *p* = <0.001), PER2 (R^2^ = 0.739; *p* = <0.001), RORA (R^2^ = 0.622; *p* = <0.001), ARNTL (R^2^ = 0.569; *p* = <0.001), CLOCK (R^2^ = 0.551; *p* = <0.001), CRY (R^2^ = 0.703; *p* = <0.001) and CRY2 (R^2^ = 0.732; *p* = <0.001).

## 4. Discussion

To the best of our knowledge, this is the first study to offer clinical evidence that HIF-1α and a wide range of clock genes expression are strongly associated in humans. Although there is still a wide gap of knowledge about the mechanisms that support this relationship, the molecular interaction between hypoxia-inducible factors and circadian clock transcription has been previously described in animal models and in vivo studies [22]. In this regard, in mice, hypoxia upregulates protein levels of PER1 and CLOCK genes decreasing its proteolytic degradation through a protein-protein interaction between HIF-1α and PER1 [23]. Similarly, HIF-1α cooperates with BMAL1/MOP3 and CLOCK to regulate gene expression in a cell line of mouse neuroblastoma [24]. On the other hand, the negative circadian regulator CRY1, but not CRY2, reduces HIF-1α half-life and affects the hypoxia response in mice [25]. Our data reinforce the hypothesis that a crosstalk between hypoxia and circadian signaling pathways exist in a HIF-1α dependent fashion [26]. Reciprocally, PER2 and CRY1 are capable to periodically inhibit the transcriptional upregulation of vascular endothelial growth factor during hypoxia, contributing to the daytime circadian fluctuation of anaerobic glycolysis in muscle mice [27,28]. This data also explains the daily rhythms in tissue oxygenation detected in the blood and tissues from rodents [29]. Altogether, our results also support the existence of a hitherto little-known interconnection that affects metabolic function in stressed conditions such as continuous nocturnal hypoxia.

**Figure 3:**
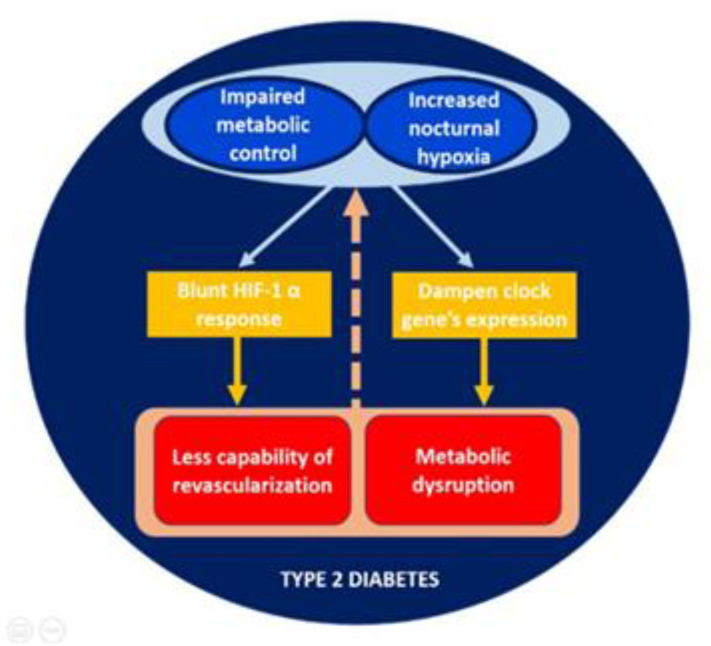
Proposed vicious circle in the relation between nocturnal sleep breathing disorders and disruption of circadian rhythmicity in patients with type 2 diabetes.

The impaired HIF-1α gene expression in patients with type 2 diabetes in comparison with controls merits further attention. Previous experimental data has revealed a compromised cellular response to hypoxia in type 2 diabetes [8,9]. In our study, the decrease in HIF-1α gene expression observed in type 2 diabetes was exacerbated in those patients with worse metabolic control. This data is relevant for those who take care of patients with type 2 diabetes, as diabetes has been established as an independent risk factor for higher rates of sleep apnea leading to nocturnal hypoxemia [3,4]. Although mechanisms contributing to this negative effect are unknown, it is palpable that the proper operating oxygen-sensing pathways are critical for adaptation to variations in oxygen availability. Therefore, our results may suggest the existence of a vicious circle in which poor metabolic control and nocturnal sleep breathing disorders collaborate to promote hypoxic dependent complications in type 2 diabetes (Figure 3).

Type 2 diabetes was also associated with a significant reduction in the expression of a great variety of clock genes. Our results are in concordance with those published by Ando H et al. in a smaller group of patients, in which mRNA expression pattern of BMAL1, PER1, PER2 and PER3 were significantly lower in patients with diabetes than in healthy controls [30]. In this study, the transcript expression of the same genes was inversely correlated with HbA1c levels [30]. It is well known, from the study of work rotating night shifts, that the disruption of circadian rhythmicity is strongly associated with the appearance of metabolic alterations such as obesity, hypertension, and metabolic syndrome [31,32]. Whether these metabolic disruptions are perpetuated by chronic hyperglycemia or sleep breathing disorders remains to be elucidated. Similarly, the combined downregulation in the expressions of CRY1 and PER3 at midnight predicted the severity of the disease in patients with obstructive sleep apnea/hypopnea syndrome [18]. Additionally, in patients with type 2 diabetes, those with the poorest sleep quality showed a dampened mRNA expression of clock genes such as BMAL1 and PER1 [33]. Therefore, we propose that the disorder in nocturnal breathing that characterize the sleep of patients with type 2 diabetes could alter the circadian rhythm (Figure 3). It is also important to emphasize that these alterations are closely associated with the degree of metabolic control, leading to consider the potential effect that the improvement of glycemic control can exert on the recovery expression of HIF-1α and clock genes. Similarly, and following this bidirectional pattern, it is plausible to think that the treatment of nocturnal hypoxemia with CPAP can partially restore the impaired hypoxic pathways and improve the clock genes transcription. However, Moreira *et al*. failed to find improvement in CLOCK expression with CPAP treatment in 17 men with severe SAHOS without type 2 diabetes [34]. This result could suggest that other mechanisms beyond hypoxia and hyperglycemia are involved in the complex regulation of the clock gene system.

There are specific constraints that should be considered in evaluating the results of our study. First, we have not measured the level of HIF protein. Therefore, our results showing the activation of HIF depending on the level of mRNA should be taken with caution since the expression of HIF1 gene does not necessarily coincide with its activation [35]. While a variety of feedback loops have been defined to enhance and ameliorate HIF signaling, the overall direction of the effect and its magnitude are difficult to confirm without knowing the final protein concentration. Second, this is a cross sectional study whereby causality cannot be determined. Third, we evaluated a relatively small number of individuals with type 2 diabetes which means that no definite outcomes for daily clinical practice can be extrapolated to the general population. Fourth, we have not considered the role of antidiabetic therapies in our study. Metformin, the most widely prescribed *insulin*-sensitizing agent in current clinical use, reverses the inhibitory effect of 11mM glucose on clock genes as BMAL1, CLOCK and PER2a in pancreatic alpha-cells [36]. Similarly, Barnea M et al. have shown that metformin enhances the CLOCK: BMAL1 expression in liver and muscle from young normoweight mice without diabetes [37]. Therefore, larger studies with a longer follow-up are required to confirm and extend our results, as well as to go deeper in the potential impact of different antidiabetic drugs. Finally, data related to physical activity were not specifically included in the analysis of the results. Since endurance training in skeletal muscle attenuates HIF-1α response to exercise [38] it could influence our results. However, the high proportion of sedentarism in patients included in the study makes this possibility negligible

## 5. Conclusions

In conclusion, this study demonstrates that type 2 diabetes alters the gene expression of HIF-1α and clock genes, which correlates with the degree of metabolic control. Further studies to better understand the link between oxygen homeostasis and chronobiological pattern may provide new insights into the involved mechanisms and innovative therapeutic strategies for patients with type 2 diabetes.

## Figures and Tables

**Table 1:** Clinical and anthropometric data of the study population according to the presence of type 2 diabetes.

	Type 2 Diabetes	Non-Type 2 Diabetes	*p*
*n*	62	67	-
Age (Years)	57.3 ± 10.0	58.8 ± 9.4	0.381
Women, *n* (%)	32 (51.6)	17 (25.3)	0.003
BMI (Kg/m^2^)	33.6 ± 6.2	28.6 ± 6.6	<0.001
Hba1c (%)	8.4 ± 1.8	5.4 ± 0.3	<0.001
Hba1c (mmol/mol)	68.9 ± 19.8	35.6 ± 3.7	<0.001
Fasting Glucose (mmol/L)	8.6 ± 3.1	4.9 ± 1.3	<0.001
Total Cholesterol (mmol/L)	45.7 ± 10.6	53.5 ± 15.8	0.173
HDL Cholesterol (mmol/L)	11.7 ± 3.1	14.8 ± 5.2	0.018
LDL Cholesterol (mmol/L)	26.8 ± 9.1	35.3 ± 7.5	0.426
Triglycerides (mmol/L)	2.1 ± 1.2	1.3 ± 0.7	0.006
Hypertension, *n* (%)	50 (80.6)	13 (24.0)	<0.001
Smoking Habit, *n* (%)	23 (37.0)	22 (32.8)	0.348
Cardiovascular Disease, *n* (%)	1 (0.01)	2 (0.002)	0.518
Retinopathy, *n* (%)	16 (25.8)	-	<0.001
Nephropathy, *n* (%)	20 (32.2)	-	<0.001
Lactate (uM/L)	2102.1 ± 688.2	1730.4 ± 694.4	0.013
Pyruvate (uM/L)	61.9 ± 25.6	50.3 ± 23.1	0.026

Data are means ± SD, median (interquartile range) or n (percentage). BMI: body mass index; HbA1c: glycated hemoglobin; HDL: high-density lipoprotein; LDL: low-density lipoprotein.

**Table 2:** Linear correlations between the expression of the 8 clock genes assessed in our study and glycated hemoglobin and HIF-1α gene expression.

	HbA1c		HIF-1α	
	r	*p*	r	*p*
HIF-1α	−0.358	<0.001	-	-
HIF-2α	−0.168	0.058	0.615	< 0.001
PER1	−0.313	<0.001	0.833	<0.001
PER2	−0.438	<0.001	0.868	<0.001
PER3	−0.328	<0.001	0.657	<0.001
RORA	−0.256	0.003	0.758	<0.001
ARNTL	−0.293	0.001	0.776	<0.001
CLOCK	−0.327	<0.001	0.814	<0.001
CRY1	−0.301	0.001	0.834	<0.001
CRY2	−0.279	0.001	0.817	<0.001

HIF: Hypoxia-Inducible Factor; PER: period homolog proteins; RORA: Retinoid-related orphan receptor alpha; ARNTL: Aryl hydrocarbon receptor nuclear translocator-like protein-1; CLOCK: Circadian locomotor output cycles kaput; CRY: Cryptochrome proteins; HbA1c: glycated hemoglobin.

**Table 3:** Variables independently related to HIF-1α gene expression in the multiple regression analysis (stepwise method) without (model 1) or with (model 2) the inclusion of clock genes as independent variables.

**Model 1**	**β**	**Beta 95% CI**	***p***
Hba1c (%)	−0.226	−0.323 (−0.344 to −0.109)	<0.001
Gender (Female/Male)	0.069	-	0.433
BMI (Kg/m^2^)	0.041	-	0.635
Age (Years)	0.013	-	0.878
Constant	-	3.363 (2.523 to 4.204)	<0.001
R^2^ = 0.104			
**Model 2**	**Β**	**Beta 95% CI**	***p***
PER1	0.519	0.307 (0.262 to 0.351)	<0.001
PER2	0.254	0.345 (0.125 to 0.566)	0.002
CRY2	0.227	0.540 (0.198 to 0.882)	0.002
CLOCK	0.192	0.328 (0.075 to 0.580)	0.011
PER3	−0.160	−0.363 (−0.581 to −0.145)	0.001
Gender (Female/Male)	0.051	-	0.058
RORA	−0.075	-	0.221
Hba1c (%)	−0.033	-	0.245
CRY1	−0.084	-	0.372
BMI (Kg/m^2^)	−0.008	-	0.750
ARNTL	−0.017	-	0.823
Age (Years)	0.000	-	1.000
Constant	-	−0.223 (−0.429 to −0.017)	0.034
R^2^ = 0.920			

β: standardized coefficient; Beta: non-standardized coefficient; HbA1c: glycated hemoglobin; BMI: body mass index; CI: confidence interval; HIF: Hypoxia-Inducible Factor; PER: period homolog proteins; RORA: Retinoid-related orphan receptor alpha; ARNTL: Aryl hydrocarbon receptor nuclear translocator-like protein-1; CLOCK: Circadian locomotor output cycles kaput; CRY: Cryptochrome proteins.

## References

[1] Sampol G., Lecube A. (2012). Type 2 diabetes and the lung: A bidirectional relationship. Endocrinol. Nutr..

[2] Aurora R.N., Punjabi N.M. (2013). Obstructive sleep apnoea and type 2 diabetes mellitus: A bidirectional association. Lancet Respir. Med..

[3] Lecube A., Sampol G., Lloberes P., Romero O., Mesa J., Hernández C., Simó R. (2009). Diabetes is an independent risk factor for severe nocturnal hypoxemia in obese patients. A case-control study. PLoS ONE.

[4] Lecube A., Simó R., Pallayova M., Punjabi N.M., López-Cano C., Turino C., Hernández C., Barbé F. (2017). Pulmonary function and sleep breathing: Two new targets for type 2 diabetes care. Endocr. Rev..

[5] López-Cano C., Gutiérrez-Carrasquilla L., Sánchez E., González J., Yeramian A., Martí R., Hernández M., Cao G., Ribelles M., Gómez X. (2019). Sympathetic hyperactivity and sleep disorders in individuals with type 2 diabetes. Front. Endocrinol. Lausanne.

[6] Pugh C.W., Ratcliffe P.J. (2017). New horizons in hypoxia signaling pathways. Exp. Cell Res..

[7] Wang G.L., Jiang B.H., Rue E.A., Semenza G.L. (1995). Hypoxia inducible factor 1 is a basic-helix-loop-helix-PAS heterodimer regulated by cellular O2 tension. Proc. Natl. Acad. Sci. USA.

[8] Kim J.W., Tchernyshyov I., Semenza G.L., Dang C.V. (2006). HIF-1-mediated expression of pyruvate dehydrogenase kinase: A metabolic switch required for cellular adaptation to hypoxia. Cell Metab..

[9] Cheng K., Ho K., Stokes R., Scott C., Lau S.M., Hawthorne W.J., O’Connell P.J., Loudovaris T., Kay T.W., Kulkarni R.N. (2010). Hypoxia-inducible factor-1alpha regulates beta cell function in mouse and human islets. J. Clin. Investig..

[10] Roenneberg T., Merrow M. (2016). The circadian clock and human health. Curr. Biol..

[11] Kalsbeek A., Yi C.X., La Fleur S.E., Fliers E. (2010). The hypothalamic clock and its control of glucose homeostasis. Trends Endocrinol. Metab..

[12] Brown S.A. (2014). Circadian clock-mediated control of stem cell division and differentiation: Beyond night and day. Development.

[13] Huang W., Ramsey K.M., Marcheva B., Bass J. (2011). Circadian rhythms, sleep, and metabolism. J. Clin. Investig..

[14] Poggiogalle E., Jamshed H., Peterson C.M. (2018). Circadian regulation of glucose, lipid, and energy metabolism in humans. Metabolism.

[15] Vieira E., Burris T.P., Quesada I. (2014). Clock genes, pancreatic function, and diabetes. Trends Mol. Med..

[16] Froy O., Garaulet M. (2018). The circadian clock in white and brown adipose tissue: Mechanistic, endocrine, and clinical aspects. Endocronol. Rev..

[17] Stenvers D.J., Scheer F.A.J.L., Schrauwen P., La Fleur S.E., Kalsbeek A. (2019). Circadian clocks and insulin resistance. Nat. Rev. Endocrinol..

[18] Yang M.Y., Lin P.W., Lin H.C., Lin P.M., Chen I.Y., Friedman M., Hung C.F., Salapatas A.M., Lin M.C., Lin S.F. (2019). Alternations of circadian clock genes expression and oscillation in obstructive sleep apnea. J. Clin. Med..

[19] Butler M.P., Smales C., Wu H., Hussain M.V., Mohamed Y.A., Morimoto M., Shea S.A. (2015). The circadian system contributes to apnea lengthening across the night in obstructive sleep apnea. Sleep.

[20] Amplification Efficiency of TaqMan Gene Expression Assays. Applied Biosystems Application Note Can Be Found Online. http://docs.appliedbiosystems.com/pebiodocs/00113186.pdf.

[21] Bustin S.A., Benes V., Garson J.A., Hellemans J., Huggett J., Kubista M., Mueller R., Nolan T., Pfaffl M.W., Shipley G.L. (2009). The MIQE guidelines: Minimum information for publication of quantitative real-time PCR experiments. Clin. Chem..

[22] von Allmen D.C., Francey L.J., Rogers G.M., Ruben M.D., Cohen A.P., Wu G., Schmidt R.E., Ishman S.L., Amin R.S., Hogenesch J.B. (2018). Circadian dysregulation: The next frontier in obstructive sleep apnea research. Otolaryngol. Head Neck Surg..

[23] Chilov D., Hofer T., Bauer C., Wenger R.H., Gassmann M. (2001). Hypoxia affects expression of circadian genes PER1 and CLOCK in mouse brain. FASEB J..

[24] Ghorbel M.T., Coulson J.M., Murphy D. (2003). Cross-talk between hypoxic and circadian pathways: Cooperative roles for hypoxia-inducible factor 1alpha and CLOCK in transcriptional activation of the vasopressin gene. Mol. Cell Neurosci..

[25] Dimova E.Y., Jakupovic M., Kubaichuk K., Mennerich D., Chi T.F., Tamanini F., Oklejewicz M., Hänig J., Byts N., Mäkelä K.A. (2019). The circadian clock protein cry1 is a negative regulator of HIF-1α. iScience.

[26] Choudhry H., Harris A.L. (2018). Advances in hypoxia-inducible factor biology. Cell Metab..

[27] Peek C.B., Levine D.C., Cedernaes J., Taguchi A., Kobayashi Y., Tsai S.J., Bonar N.A., McNulty M.R., Ramsey K.M., Bass J. (2017). Circadian clock interaction with HIF1a mediates oxygenic metabolism and anaerobic glycolysis in skeletal muscle. Cell Metab..

[28] Wu Y., Tang D., Liu N., Xiong W., Huang H., Li Y., Ma Z., Zhao H., Chen P., Qi X. (2017). Reciprocal regulation between the circadian clock and hypoxia signaling at the genome level in mammals. Cell Metab..

[29] Adamovich Y., Ladeuix B., Golik M., Koeners M.P., Asher G. (2017). Rhythmic oxygen levels reset circadian clocks through HIF1a. Cell Metab..

[30] Ando H., Takamura T., Matsuzawa-Nagata N., Shima K.R., Eto T., Misu H., Shiramoto M., Tsuru T., Irie S., Fujimura A. (2009). Clock gene expression in peripheral leucocytes of patients with type 2 diabetes. Diabetologia.

[31] Eckel-Mahan K., Sassone-Corsi P. (2013). Metabolism and the circadian clock converge. Physiol. Rev..

[32] Kalsbeek A., Fliers E. (2017). Circadian and endocrine rhythms. Best Pract. Res. Clin. Endocrinol. Metab..

[33] Huang Y., Wang H., Li Y., Tao X., Sun J. (2017). Poor sleep quality is associated with dawn phenomenon and impaired circadian clock gene expression in subjects with type 2 diabetes mellitus. Int. J. Endocrinol..

[34] Moreira S., Rodrigues R., Barros A., Pejanovic N., Neves-Costa A., Pedroso D., Pereira C., Fernandes D., Valenca Rodrigues J., Barbara C. (2017). Changes in expression of the clock gene in obstructive sleep apnea syndrome patients are not reverted by continuous positive airway pressure treatment. Front. Med. Lausanne.

[35] Pugh C.W. (2016). Modulation of the hypoxic response. Adv. Exp. Med. Biol..

[36] Vieira E., Marroquí L., Figueroa A.L., Merino B., Fernandez-Ruiz R., Nadal A., Burris T.P., Gomis R., Quesada I. (2013). Involvement of the clock gene rev-erb alpha in the regulation of glucagon secretion in pancreatic alpha-cells. PLoS ONE.

[37] Barnea M., Haviv L., Gutman R., Chapnik N., Madar Z., Froy O. (2012). Metformin affects the circadian clock and metabolic rhythms in a tissue-specific manner. BiochimBiophys. Acta.

[38] Hawley J.A., Lundby C., Cotter J.D., Burke L.M. (2018). Maximizing cellular adaptation to endurance exercise in skeletal muscle. Cell Metab..

